# Evidence-based health care for all: support from the Brazilian Ministry of Health

**DOI:** 10.1590/S1516-31802007000300001

**Published:** 2007-05-03

As you well know, after Semmelweis made the fundamental discovery of the cause of puerperal infection (contamination from doctors’ hands), he died as a “madman” in a hospital, tormented by the lack of comprehension of his findings. His colleagues, who had been performing puerperal examinations without antiseptic procedures for so long, did not believe the new evidence. Even today, it is known that not washing hands, stethoscopes, etc, between examining one patient and the next is one of the most incontestable causes of hospital infections.

Infections resulting from contamination with catheters in intensive care are common, potentially lethal and cause great human and financial losses. A study published in the New England Journal of Medicine^1^ showed that four simple evidence-based procedures reduced bloodstream infections due to catheters by 66%:

1. washing hands;
2. use of complete barriers when inserting central catheters;
3. asepsis with chlorhexidine instead of iodine, on the skin in the area to be punctured;
4. avoidance of the use of catheters in the femoral region and, whenever possible, removal of unnecessary catheters.

In parallel with this, continuing preventive education campaigns on the harm done by catheter infections have been implemented. These are simple measures that will certainly save many lives and avoid great suffering and waste of resources (around U$ 45,000.00 per case in the United States^2^).

With regard to pregnant women's health, it should be highlighted that the results from the Brazilian Ministry of Health's Maternal Mortality Prevention Pact were released in the Federal University of São Paulo (Unifesp), under the coordination of the Brazilian Cochrane Center. On that occasion, we had the honor of welcoming the Minister of Health, Prof. Dr. José Gomes Temporão, the Special Minister for Women's Policies, Nilcéa Freire, the President of the House of Representatives, Arlindo Chinaglia, and the President of the Republic, Luís Inácio Lula da Silva. Also on that occasion, Minister Temporão expressed his desire for his administration to be conducted based on evidence, with collaboration from the Brazilian Cochrane Center. President Lula emphasized the need to recognize and combat the causes of maternal mortality, which in Brazil is very high (around 73 cases per 100,000 live births).^3^

Minister Temporão accepted an invitation to make the opening speech at the World Congress of Evidence-Based Healthcare, to be held in São Paulo from October 23 to 27, 2007: www.cochranecolloquium.info. The scientific program, which is now ready, will include participation by specialists from around 80 countries, in 60 workshops, 180 presentations of scientific studies, 12 special sessions and plenary sessions to bring the evidence together. In addition to all of this, there will be unmissable social activities, shows featuring the “Brazilian popular music” style (MPB), a tropical dinner-dance and a soccer “match of the legends” of the Brazilian national team in the Pacaembu stadium, organized by Dr. Sócrates de Oliveira. For further information, access: www.cochranecolloquium.info.
